# Topographical Anatomy of the Adductor Muscle Group in the Albino Rat (*Rattus norvegicus*)

**DOI:** 10.3390/life13102096

**Published:** 2023-10-21

**Authors:** Bettina Pretterklieber, Michael L. Pretterklieber, Katharina Kerschan-Schindl

**Affiliations:** 1 Division of Macroscopic and Clinical Anatomy, Gottfried Schatz Research Center, Medical University of Graz, 8036 Graz, Austria; michael.pretterklieber@medunigraz.at; 2 Division of Anatomy, Center for Anatomy and Cell Biology, Medical University of Vienna, 1090 Vienna, Austria; 3 Department of Physical Medicine, Rehabilitation and Occupational Medicine, Medical University of Vienna, 1090 Vienna, Austria; katharina.kerschan-schindl@meduniwien.ac.at

**Keywords:** comparative myology, hip muscles, musculoskeletal system, obturator intermedius muscle, obturator nerve

## Abstract

In comparative anatomy, the adductor muscles are said to be quite variable and to often cause difficulty in separation. The arrangement of these muscles and the possible occurrence of the adductor minimus and obturator intermedius muscles in the albino rat has not been investigated. The aim of this study was to accurately describe the adductor muscles in the albino rat (*Rattus norvegicus*). We hypothesized that all adductor muscles are constantly present and can be separated in a constant manner, and that the adductor minimus and obturator intermedius muscles are constant structures. Both pelvic limbs of 30 formalin-embalmed male albino rats were carefully dissected. The identification of the individual muscles was made based on their position in relation to the two branches of the obturator nerve and by comparing our results with previous findings in other species including humans. All examined rats had two gracilis muscles. The adductor longus muscle was the most superficial and smallest individual. The adductor brevis split into two parts of insertion—the femoral and genicular parts. The adductor magnus and minimus muscles could be separated constantly. The obturator intermedius muscle was a constant structure next to the obturator externus muscle. The adductor muscles of the albino rat were constantly separable and could be clearly assigned to their names. Further research is needed to investigate these muscles, especially the obturator intermedius muscle, in other species including humans.

## 1. Introduction

In all mammals, including humans, the adductor muscle group is situated in the medial aspect of the femoral region [1,2,3,4,5]. This muscle group is derived from the ventral lumbar group, which is innervated by the obturator nerve. It includes the adductor muscles proper (adductor longus, brevis, magnus, and minimus muscles), and the gracilis and obturator externus muscles [1,2]. Even though the pectineus muscle is often described as an adductor muscle [6,7], this seems to be incorrect regarding its phylogenesis and ontogenesis [1,2,8].

In many animals, as for example in ungulates or some other domestic mammals and anthropoid apes, the gracilis muscle forms one singular broad muscle plate [2,9,10,11]. However, in mammals, especially rodents, it can also be partially or completely duplicated [1,2,6,7,8,12]. However, this seems to be a secondary process, since it is known for the mouse that the originally single-headed gracilis muscle divides only during further embryogenesis [1]. The adductor muscles proper are very variable and are often said to cause difficulty in separating into individual parts [2]. In urodeles, turtles, and saurians, it is still a uniform muscle mass. In crocodilians and birds, this muscle already splits into two parts. In monotremes, there are usually three adductor muscles [7]. In domestic mammals (horse, pig, dog), sometimes only a single adductor muscle mass is described [9]. Often two or three individual muscles are mentioned [11,13,14,15,16], which are usually named according to human anatomy [2]. An additional adductor minimus muscle is described in anthropoids [10]. In rodents, it can sometimes split off from the adductor magnus [17]. The obturator externus muscle seems to be relatively constant in all mammals [1,2,6,8,9,10,11,12,17,18,19,20]. In anurans and monotremes, it consists of two, in saurians even of three portions [7]. It tends to be divided by the course of the obturator nerve in many mammals (especially primates and rodents). Then, its ventral portion is the so-called obturator intermedius (tertius) muscle [2,7].

The adductor muscles are partly well described in humans and non-human primates [3,5,10,21,22,23]. For mammals, it is generally said that they should not be separated uniformly. Therefore, there are numerous different descriptions [2,7,9,11,24,25]. Although there are some detailed descriptions of these muscles in other rodents [8,12,17], information on this muscle group in the albino rat (*Rattus norvegicus*) is rather cursory [18,26]. In addition, no descriptions of rodents even in detailed books of comparative anatomy [2,7] exist, which would be helpful for these muscles’ exact dissection in the rat. Until now, the possible occurrence of the adductor minimus and obturator intermedius muscles in the albino rat has not been investigated.

The aim of this study was to provide an accurate description of the topography of the adductor muscle group in the albino rat. Furthermore, we wanted to discuss the similarities and differences observed in the albino rat and other mammals, especially humans. The comparison with human anatomy should show whether this muscle group is arranged similarly, despite the different gait pattern (quadrupedal versus bipedal). If so, further studies on hip muscles, such as muscle fiber typing or number of muscle spindles, could be performed on the rat model.

We hypothesize that in the albino rat all muscles of the adductor group are constantly present and can always be separated in the same way. The gracilis muscle consists of two parts, as in other rodents. In addition, the adductor minimus and obturator intermedius muscles are constant structures.

## 2. Material and Methods

Both pelvic limbs of 30 adult (12 to 14 weeks old) male albino rats (24 Sprague Dawley, 6 Wistar) were carefully dissected. The rats had been used for other studies not involving the musculoskeletal system. Before being placed at our disposal they had been already sacrificed. Thus, for the present study, no approval of the local animal research ethics committee was necessary. Especially the trunk and pelvic limbs of the rats were skinned; the organs including the external genitals were carefully removed. The specimens were then embalmed by immersion in a 2% solution of non-buffered formalin for several weeks. Following fixation, the specimens were stored in a low-percent solution of phenol. The same rats were used for the dissection of other muscle groups. The results of the ischiotrochanteric muscle group have been recently published [27].

Careful stratigraphic dissection was performed, similar to the procedure in human specimens, using a magnification lens. As providing a detailed dissection guide was one of the aims of this study, the dissection protocol is given as part of the results. Photographs were taken using a digital reflex camera (Canon EOS 5D Mark II, Canon Inc., Tokyo, Japan). All terms used in this study are following the English version of the Nomina anatomica veterinaria [13,28]. In the absence of official terms given by the veterinary anatomy, the terms have been used according to the English version of the Terminologia anatomica [29,30,31]. In exceptional instances, other definitions were applied. The naming of the individual muscles was based on their position in relation to each other and to the two branches of the obturator nerve. The latter was identified based on its origin from the lumbar plexus, and its course along the lateral wall of the lesser pelvis and through the obturator canal.

**Figure 1:**
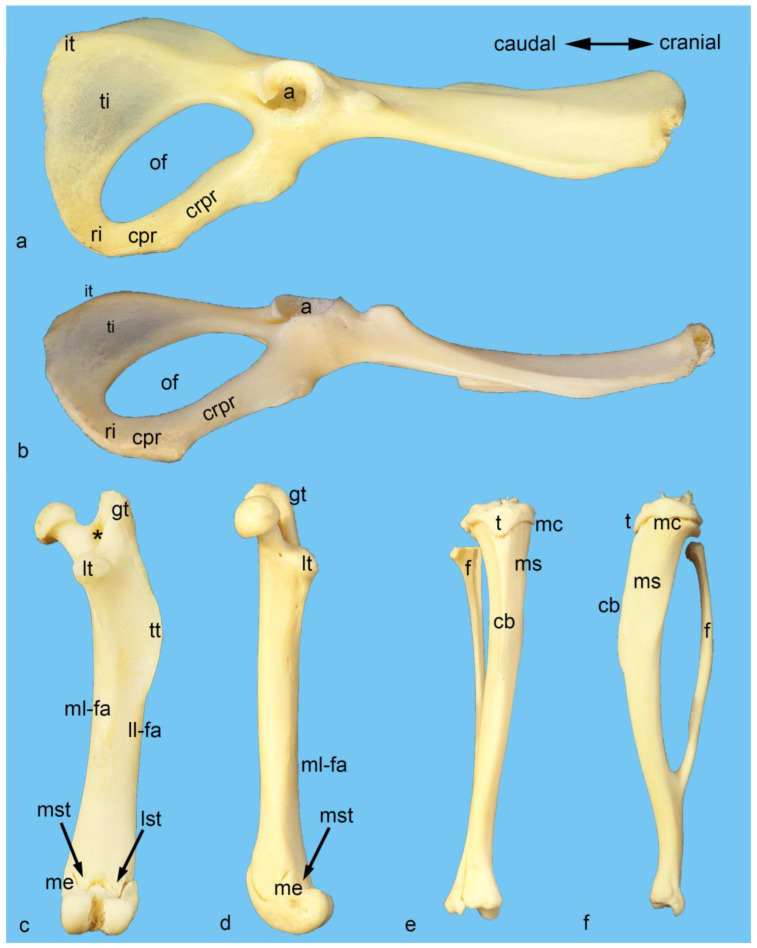
Bony structures important for this study. Photos of macerated bones from a male albino rat were taken to provide an overview of the bony structures important for this study. Isolated right hip bone from the lateral (**a**) and ventral (**b**) aspects. Isolated right femur from the caudal (**c**) and medial (**d**) aspects. Isolated right tibia and fibula from the cranial (**e**) and medial (**f**) aspects. a = acetabulum, cb = cranial border of the tibia, cpr = caudal pubic ramus, crpr = cranial pubic ramus, f = fibula, gt = greater trochanter, it = ischial tuber, ll-fa = lateral lip of facies aspera, lst = lateral supracondylar tuberosity, lt = lesser trochanter, mc = medial (tibial) condyle, me = medial (femoral) epicondyle, ml-fa = medial lip of facies aspera, ms = medial surface of tibia, mst = medial supracondylar tuberosity, of = obturator foramen, ri = ramus of ischium, t = tibial tuberostiy, ti = tabula of ischium, tt = third trochanter, asterisk = trochanteric fossa.

**Figure 2:**
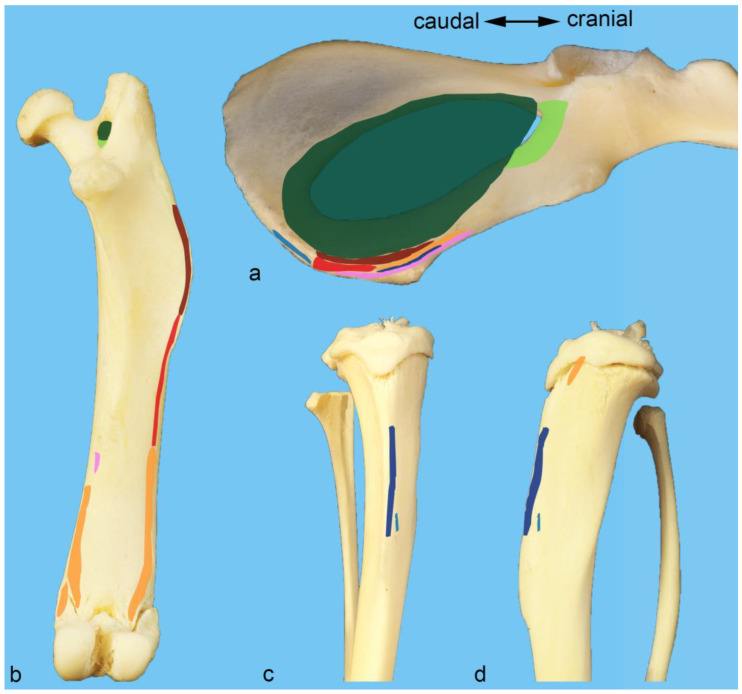
Muscle attachments of the adductor group. Origin and insertion areas of the adductor muscles are color-coded on photographs of isolated macerated bones from a male albino rat. The caudal half of the hip bone is shown from the ventral aspect (**a**). The femur is shown from the caudal aspect (**b**). The proximal part of the tibia is shown from the cranial (**c**) and medial (**d**) aspects. Color key: dark blue—cranial gracilis muscle, light blue—caudal gracilis muscle, pink—adductor longus muscle, orange—adductor brevis muscle, light red—adductor magnus muscle, dark red—adductor minimus muscle, dark green—obturator externus muscle, light green—obturator intermedius muscle.

To give an overview of the bony structures important for this study, photographs were taken from macerated bones from one additional male albino rat (Figure 1). The origin and attachment areas of all the muscles examined were color-coded in some of these photos using Adobe Photoshop CS6 (Figure 2). 

To compare the adductor muscles of the albino rat with other species, general comparative and veterinary textbooks and original research were studied. The comparison with human anatomy is mainly based on detailed human anatomical textbooks, some studies concerning the anatomy of the hip muscles, and our own years of experience in the dissecting room. The respective references are given with the corresponding statements in the discussion.

## 3. Results

In all cases, it was possible to identify constantly all muscles of the adductor muscle group by their relation to the two branches of the obturator nerve. Rarely, remarkable variations were found to be present. The systematic details of the investigated muscles are summarized in Table 1.

### 3.1. Cranial Gracilis Muscle

**Figure 3:**
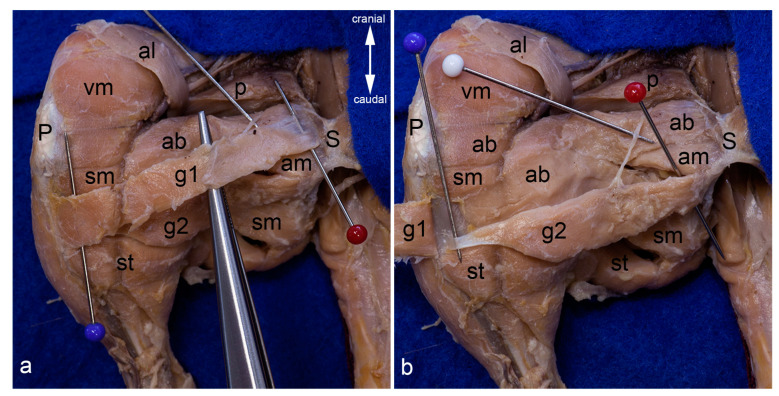
Gracilis muscles. The adductor longus muscle was detached from its origin and reflected towards its insertion. Thereby its innervating twig from the cranial branch of the obturator nerve has been cut. The cranial gracilis muscle can be observed in toto from the medial aspect of the thigh (**a**). The cranial gracilis muscle was also detached from its origin and reflected laterally after transecting its innervating twig from the cranial branch of the obturator nerve. The caudal gracilis muscle can be observed in toto (**b**). In both pictures, the thigh was abducted in the hip joint to show the medial aspect of the thigh. The red pins highlight the origin, the blue pins the insertion, and the white pins the innervation of the gracilis muscles. ab = adductor brevis muscle, al = adductor longus muscle, am = adductor magnus muscle, g1 = cranial gracilis muscle, g2 = caudal gracilis muscle, P = patella/patellar ligament, p = pectineus muscle, S = pelvic symphysis, sm = semimembranosus muscle, st = semitendinosus muscle, vm = vastus medialis muscle.

The cranial gracilis muscle was a long flat muscle (Figure 3a). Its proximal portion was partly covered by the course of the adductor longus muscle. It arose with a thin aponeurosis from the cranial and caudal pubic rami, and inserted fleshy on the cranial border of the tibia between the tibial tuberosity and the insertion of the semitendinosus muscle (Figure 2 and Figure 3a). A twig from the cranial branch of the obturator nerve entered the muscle on its deep surface (Figure 3a). 

### 3.2. Caudal Gracilis Muscle

The caudal gracilis muscle was also a long but somewhat thicker muscle (Figure 3b). It narrowed towards its distal end, where it was covered by the broad distal portion of the cranial gracilis muscle (Figure 3). It originated from the ramus of ischium between the adductor magnus and semimembranosus muscles, and showed a tendinous insertion on the medial surface of the tibia, just cranial to the semitendinosus muscle (Figure 2 and Figure 3b). In about one third of cases, a little bundle of its fibers continued into the deep aspect of the cranial gracilis muscle. It was innervated by the cranial branch of the obturator nerve. The supplying nerve branch entered the muscle on its cranial border (Figure 3b).

### 3.3. Adductor Longus Muscle

**Figure 4:**
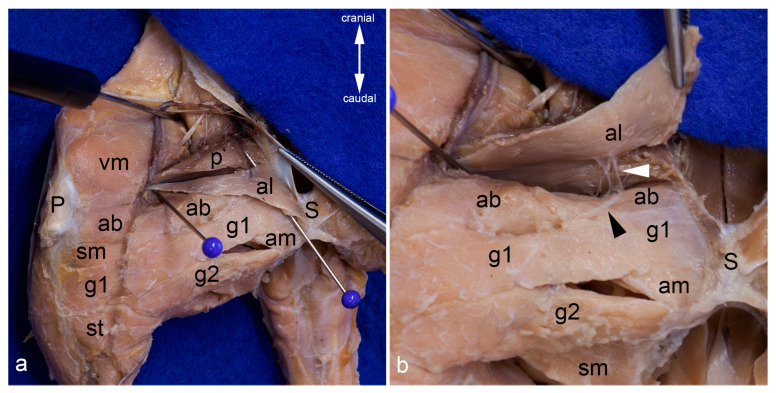
Adductor longus muscle. The adductor longus muscle is shown in situ in the medial aspect of the thigh. The blue pins highlight its attachments. The inguinal ligament is elevated by a clamp to show the broad lacunar ligament (**a**). The adductor longus muscle was mobilized from its origin to show its innervation from the cranial branch of the obturator nerve (white arrowhead). The black arrowhead indicates the course of the cranial branch of the obturator nerve (**b**). In both pictures, the thigh was abducted in the hip joint. ab = adductor brevis muscle, al = adductor longus muscle, am = adductor magnus muscle, g1 = cranial gracilis muscle g2 = caudal gracilis muscle, P = patella / patellar ligament, p = pectineus muscle, S = pelvic symphysis, sm = semimembranosus muscle, st = semitendinosus muscle, vm = vastus medialis muscle.

The adductor longus muscle was the most superficial muscle of the whole adductor group (Figure 4a). As it narrowed towards its insertion, its shape was almost triangular. In its course, it covered the proximal portions of the cranial gracilis and adductor brevis muscles. The adductor longus muscle originated from the cranial and caudal pubic rami and the ramus of ischium, and inserted on the medial lip of the facies aspera (Figure 2 and Figure 4a). Its tendon of insertion usually fused with the distal portion of the pectineus muscle. A twig from the cranial branch of the obturator nerve entered the muscle on its deep surface (Figure 4b).

### 3.4. Adductor Brevis Muscle

**Figure 5:**
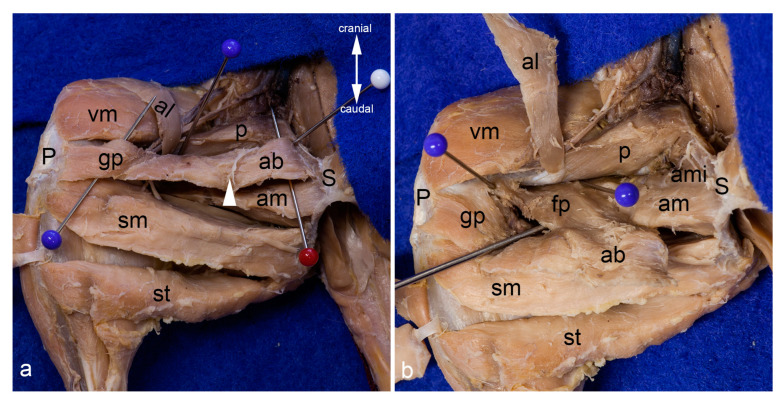
Adductor brevis muscle. The adductor longus and both gracilis muscles were reflected laterally to show the adductor brevis muscle within the medial aspect of the thigh. The red pin highlights its origin and the blue pins its insertion; only the insertion of the genicular part is visible. The white arrowhead indicates the course of the cranial branch of the obturator nerve (**a**). The adductor brevis muscle was detached from its origin and pulled distally. The insertion of its femoral part can be observed bordered by the blue pins (**b**). In both pictures, the thigh was abducted in the hip joint. ab = adductor brevis muscle, al = adductor longus muscle, am = adductor magnus muscle, ami = adductor minimus muscle, fp = femoral part of adductor brevis muscle, gp = genicular part of adductor brevis muscle, P = patella / patellar ligament, p = pectineus muscle, S = pelvic symphysis, sm = semimembranosus muscle, st = semitendinosus muscle, vm = vastus medialis muscle.

The adductor brevis muscle was relatively thick and long (Figure 5a). It was partly covered by the cranial gracilis and adductor longus muscles (Figure 4). It originated from the cranial and caudal pubic rami (Figure 2 and Figure 5a). Although it consisted of two parts, which diverged towards their insertions, they were not clearly separable near their origin (Figure 5b). About half of the muscle mass formed its femoral part. These fibers were attached to the medial lip of the facies aspera, just distal to the pectineus and adductor longus muscles, but also to the lateral lip of the facies aspera, just distal to the insertion of the adductor magnus muscle. In addition, a small portion was attached to the medial and lateral supracondylar tuberosity. The more superficial and distal portion of the adductor brevis muscle formed its genicular part. It inserted on the medial femoral epicondyle, reinforced the medial patellar retinaculum, and inserted on the medial tibial condyle (Figure 2 and Figure 5). The adductor brevis muscle was innervated by one or two small twigs from the cranial branch of the obturator nerve, which coursed close to its superficial surface. The caudal branch of the obturator nerve separated the deep aspect of the adductor brevis muscle from the adductor minimus and magnus muscles.

### 3.5. Adductor Magnus Muscle

**Figure 6:**
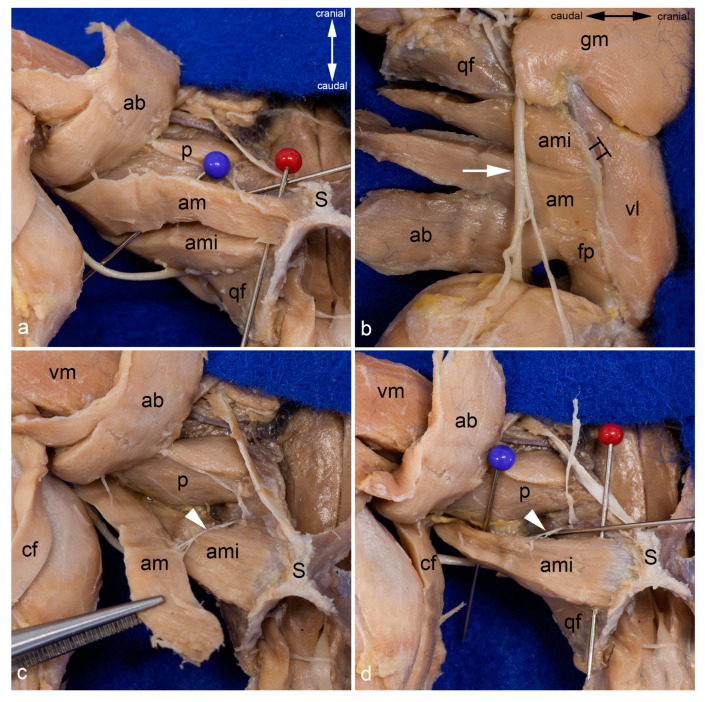
Adductor magnus and minimus muscles. The course of the adductor magnus muscle is shown from the medial aspect. The pins separate it from the adductor minimus muscle (**a**). The insertion of the adductor minimus and adductor magnus muscles can also be observed from the lateral aspect of the thigh, after releasing all hamstring muscles, the femorococcygeus muscle, and the gluteus superficialis muscle. The adductor muscles were separated from the vastus lateralis muscle by the lateral femoral intermuscular septum. The sciatic nerve (white arrow) was in contact only with the adductor minimus muscle as the caudofemoralis and biceps femoris muscles (already removed for this photograph) were situated between the nerve and the adductor magnus and brevis muscles (**b**). For this photograph the adductor magnus muscle was also released from its origin and pulled distally to show its innervation by the caudal branch of the obturator nerve (white arrowhead) (**c**). The adductor magnus muscle was removed to show the course of the adductor minimus muscle from the medial aspect. The red pin is inserted near its origin and the blue pin near its insertion. Its innervation by the caudal branch of the obturator nerve (white arrowhead) can be observed. The cranial branch of the obturator nerve was reflected cranially (**d**). In all pictures, almost all superficial muscles were detached from their origin and reflected towards their insertion or totally removed. In (**a**,**c**,**d**), the thigh was abducted in the hip joint. ab = adductor brevis muscle, am = adductor magnus muscle, ami = adductor minimus muscle, cf = caudofemoralis muscle, fp = femoral part of adductor brevis muscle, gm = gluteus medius muscle, p = pectineus muscle, qf = quadratus femoris muscle, S = pelvic symphysis, TT = third trochanter, vl = vastus lateralis muscle, vm = vastus medialis muscle.

The adductor magnus muscle was a long and relatively thick muscle (Figure 6a). It was almost completely covered by the cranial gracilis and adductor brevis muscles (Figure 4 and Figure 5). The adductor magnus muscle originated from the ramus of ischium, superficial to the adductor minimus muscle. It inserted on the caudal part of the third trochanter, just distal to the adductor minimus muscle, and on the lateral lip of the facies aspera, proximal to the femoral part of the adductor brevis muscle (Figure 2 and Figure 6a,b). There, it was separated from the vastus lateralis muscle by the lateral femoral intermuscular septum (Figure 6b). The caudal branch of the obturator nerve separated it from the adductor brevis muscle. The muscular branches entered the muscle on its cranio-lateral border (Figure 6c).

### 3.6. Adductor Minimus Muscle

The adductor minimus muscle was also a relatively thick muscle situated deep in the medial aspect of the thigh between the adductor magnus and the obturator externus muscles (Figure 6a,d). It originated from the cranial and caudal pubic rami and the ramus of ischium. The adductor minimus muscle inserted on the caudal aspect of the third trochanter, caudal to the insertion of the gluteus superficialis muscle and proximal to the adductor magnus muscle (Figure 2 and Figure 6b–d). It was innervated by a twig of the caudal branch of the obturator nerve, which entered the muscle on its cranio-lateral border (Figure 6d).

### 3.7. Obturator Externus and Intermedius Muscles

**Figure 7:**
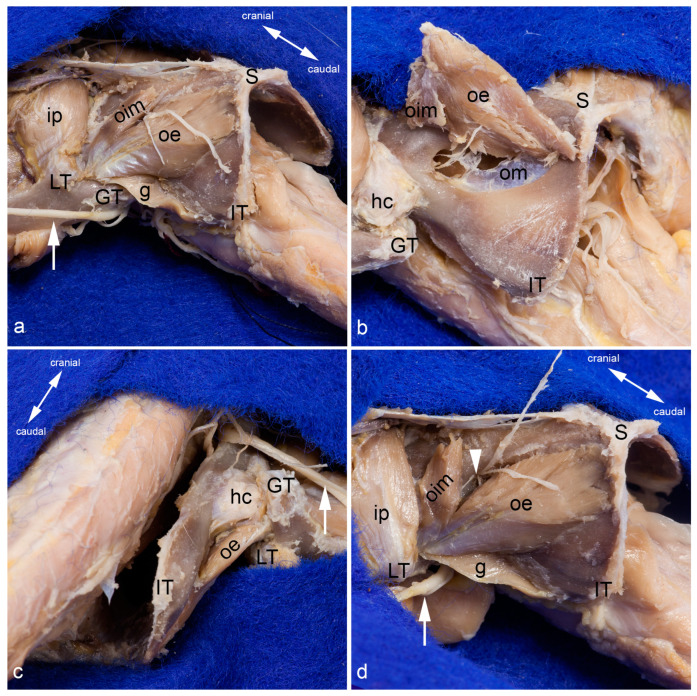
Obturator externus and intermedius muscles. The obturator externus and obturator intermedius muscles are shown from the ventrolateral aspect. Between them, the cranial and caudal branches of the obturator nerve exit (**a**). The obturator externus and intermedius muscles have been detached from their insertion and partly mobilized. Thus, their deep sites of origin can be observed from the ventrolateral aspect. The nerve twig to the obturator intermedius muscle was transected during mobilization (**b**). The course and insertion of the obturator externus muscle are shown from the dorsal aspect. The obturator intermedius muscle is located ventro-caudally and thus covered by the course of the obturator externus muscle (**c**). The obturator intermedius muscle has been partly mobilized to show the proper nerve twigs for each muscle (white arrowhead). The cranial branch of the obturator nerve was reflected cranially (**d**). In all pictures, almost all superficial muscles were detached from their origin and reflected towards their insertion or totally removed. The thigh was abducted and fully rotated laterally in (**a**,**b**,**d**). g = gemellus muscle, GT = greater trochanter, hc = capsule of the hip joint, ip= iliacus and psoas muscles, IT = ischial tuber, LT = lesser trochanter, oe = obturator externus muscle, oim = obturator intermedius muscle, om = obturator membrane, S = pelvic symphysis, white arrow = sciatic nerve.

These fan-shaped muscles were the deepest muscles of the adductor group. The obturator externus muscle was situated dorso-caudally, and the obturator intermedius muscle ventro-cranially (Figure 7a). The obturator externus muscle originated from the cranial and caudal pubic rami, from the ramus of ischium, and from the cranial margin of the tabula of ischium close to the obturator foramen. In addition, it was attached to the outer aspect of the thin obturator membrane. As the latter was fixed on the inner aspect of the obturator foramen, the muscle filled out the groove between this membrane and the outer aspect of the bony frame of the obturator foramen (Figure 2 and Figure 7a,b). The obturator intermedius muscle originated from the cranial pubic ramus. The tendons of these two muscles were more or less fused and inserted in the trochanteric fossa (Figure 2 and Figure 7a,c). In addition, they were attached to the ventro-caudal part of the fibrous capsule of the hip joint. Each of the two muscles was innervated by a proper twig of the obturator nerve, just where the latter left the obturator canal (Figure 7d).

### 3.8. Topography and Dissection Guide

**Figure 8:**
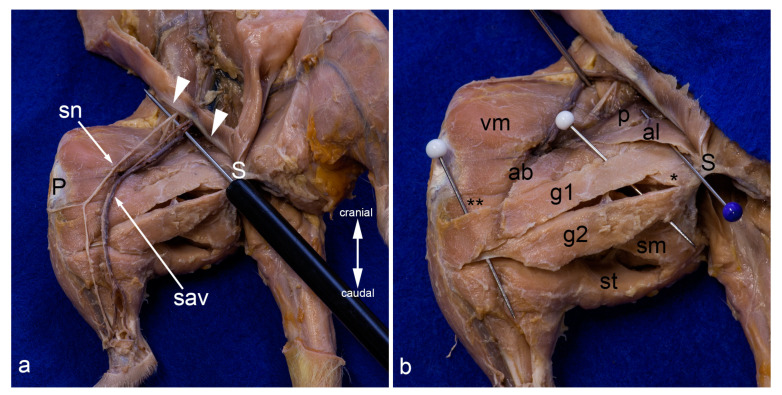
Superficial layers in the medial aspect of the thigh. On the left leg, only the skin was removed. The superficial structures gleam through the fascia lata. On the right leg, the fascia lata was totally removed. Superficial to the probe, the femoral nerve, the femoral artery, and the femoral vein (from lateral to medial) and their proximal branches (saphenous nerve, saphenous artery, and medial saphenous vein) are visible. The abdominal wall was removed except for the caudal part forming the inguinal ligament (white arrowheads) (**a**). The superficial muscles in the medial aspect of the thigh were carefully separated by blunt dissection. The saphenous nerve, the saphenous artery, and the medial saphenous vein were partially removed or displaced laterally. The blue pin indicates the most superficial muscle in this region, the adductor longus muscle. The white pins highlight the cranial gracilis and caudal gracilis muscles (**b**). In both pictures, the thigh was abducted in the hip joint to show the medial aspect of the thigh. ab= adductor brevis muscle, al = adductor longus muscle, g1 = cranial gracilis muscle, g2 = caudal gracilis muscle, P = patella/patellar ligament, p = pectineus muscle, S = pelvic symphysis, sav = saphenous artery and medial saphenous vein), sm and ** = semimembranosus muscle, sn = saphenous nerve, st = semitendinosus muscle, vm = vastus medialis muscle, * = adductor magnus muscle.

Underneath the fascia lata in the medial aspect of the thigh, the saphenous nerve, saphenous artery, and medial saphenous vein were located superficially to the first muscular layer (Figure 8a). Just caudal to the inguinal ligament, the saphenous nerve united with the femoral nerve, the saphenous artery arose from the femoral artery, and the medial saphenous vein joined the femoral vein. The femoral nerve and the femoral vessels passed through the muscular and vascular spaces, respectively. These openings connected the retroperitoneal space and the femoral region, and were bordered by the hip bone covered by the iliopsoas and pectineus muscles, the inguinal ligament, the iliopectineal arc, and the lacunar ligament. After completely removing the fascia lata, the adductor muscles could be identified and partly separated from each other, from the vastus medialis muscle, and from the medial hamstring muscles (Figure 8b). The most superficial muscle of the adductor group was the adductor longus muscle. To their widest extend, the two gracilis muscles were situated superficially too. Only the aponeurotic origin of the cranial gracilis muscle was covered by the course of the adductor longus muscle, and the distal portion of the caudal gracilis muscle was covered by the broad insertion of the cranial gracilis muscle. With its broad origin from the cranial and caudal pubic rami and the ramus of ischium, the adductor longus muscle was in close contact with the lacunar ligament (Figure 4a). With its cranio-lateral border, the muscle was adjacent to the pectineus muscle. As the adductor longus and cranial gracilis muscles diverged towards their distal attachments, the adductor brevis muscle was already partly visible between them and the vastus medialis muscle. The origin of the adductor magnus muscle was also partly visible between the origin of the cranial and caudal gracilis muscles, but in its further course, it was covered by the adductor brevis and cranial gracilis muscles. Without mobilizing any muscle, two of the hamstring muscles were also already visible in the medial aspect of the thigh. Subsequent to the origin of the adductor magnus muscle, the semimembranosus and—dorsally to it—the semitendinosus muscles were located. Although the semimembranosus muscle was mostly covered by the course of the gracilis muscles, a small portion close to its insertion on the medial tibial condyle was visible between the genicular part of the adductor brevis muscle and the cranial gracilis muscle. The semitendinosus muscle was more or less fully visible; it inserted distally to the cranial and caudal gracilis muscles on the cranial border of the tibia (Figure 8b). 

**Figure 9:**
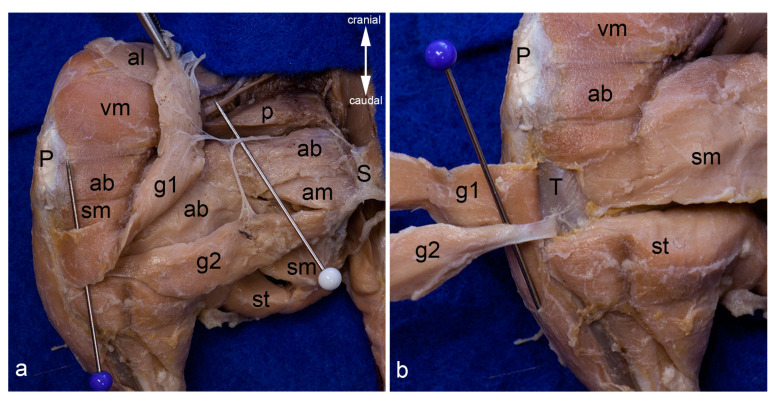
Topographical relationships of the gracilis muscles. The adductor longus and cranial gracilis muscle were mobilized from their origin and reflected laterally. The course of the cranial branch of the obturator nerve (white pin) and its twigs for the cranial gracilis and caudal gracilis muscles are visualized (**a**). Both gracilis muscles were reflected laterally to show the bursae underneath their tendons in detail (**b**). In both pictures, the thigh was abducted in the hip joint to show the medial aspect of the thigh. ab = adductor brevis muscle, al = adductor longus muscle, am = adductor magnus muscle, g1 = cranial gracilis muscle, g2 = caudal gracilis muscle, P = patella / patellar ligament, p = pectineus muscle, S = pelvic symphysis, sm = semimembranosus muscle, st = semitendinosus muscle, T = medial surface of the tibia, vm = vastus medialis muscle.

The adductor longus muscle was detached from its origin from the cranial and caudal pubic rami and the ramus of ischium, and mobilized toward its insertion on the medial lip of the facies aspera. Thereby, its innervation from the cranial branch of the obturator nerve had to be cut (Figure 4b). After releasing the origin of the adductor longus muscle, the whole area of the thin aponeurotic origin of the cranial gracilis muscle from the cranial and caudal pubic rami was visible (Figure 3a). Near its origin, the cranial gracilis muscle was situated superficial to the adductor brevis and adductor magnus muscles. Its distal portion covered the caudal gracilis and semimembranosus muscles (Figure 3a, Figure 8b, and Figure 9a). The cranial gracilis muscle was also carefully detached from its origin from the cranial and caudal pubic rami to observe the underlying muscles and almost the whole course of the cranial branch of the obturator nerve, which coursed between the adductor longus and cranial gracilis muscles (superficial), and adductor brevis muscle (deep). It innervated the adductor longus and cranial gracilis muscle on their deep surface, and coursed distally to innervate the caudal gracilis muscle (Figure 3b, Figure 4b, and Figure 9a). Finally, its terminal cutaneous branch reached the subcutaneous tissue.

The muscular twigs for both gracilis muscle were transected, and the caudal gracilis muscle was detached from its origin from the ramus of ischium. Then, both gracilis muscles were mobilized towards their insertion. The cranial gracilis muscle inserted on the cranial border of the tibia, with a bursa between its deep side and the medial surface of the tibia and the tendon of the caudal gracilis muscle. The caudal gracilis muscle inserted deep to the cranial gracilis muscle on the medial surface of the tibia, also with a bursa between its tendon and the bone (Figure 9b). 

**Figure 10:**
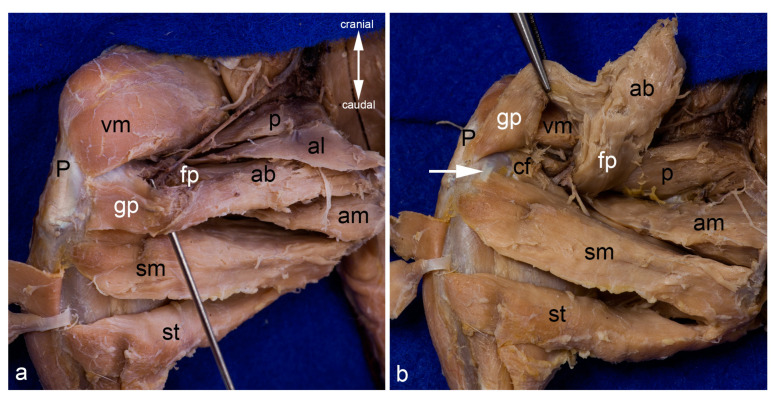
Adductor canal. The proximal end of the adductor canal between the femoral part and the genicular part of the adductor brevis muscle is indicated with the tip of the probe (**a**). The distal end of this canal between the femoral part of the adductor brevis muscle and the caudofemoralis muscle can be observed in the deep. The femoral and genicular parts of the adductor brevis muscle are visualized by detaching the muscle from its origin and reflecting it cranially. The white arrow indicates the medial collateral ligament (**b**). The cranial and caudal gracilis muscles have been detached from their origin and reflected towards their insertion in both pictures. In both pictures, the thigh was abducted in the hip joint to show the medial aspect of the thigh. ab = adductor brevis muscle (fp = femoral part, gp = genicular part), al = adductor longus muscle, am = adductor magnus muscle, cf = caudofemoralis muscle, P = Patella/patellar ligament, p = pectineus muscle, sm = semimembranosus muscle, st = semitendinosus muscle, vm = vastus medialis muscle.

Now, almost the whole course of the adductor brevis muscle was visible (Figure 5a and Figure 10a). Between the femoral part (proximal) and the genicular part (distal) of the adductor brevis muscle, the femoral vessels entered the adductor canal to reach the popliteal region (Figure 10a). The distal end of the adductor canal—the adductor hiatus—was bordered again by the femoral part of the adductor brevis muscle and by the distal portion of the caudofemoralis muscle (Figure 10b). The latter is part of the hamstring muscles, originated from the spinous process of the first coccygeal vertebra, and inserted on the medial femoral epicondyle, the medial supracondylar tuberosity, and the proximo-medial aspect of the medial fabella (unpublished data).

**Figure 11:**
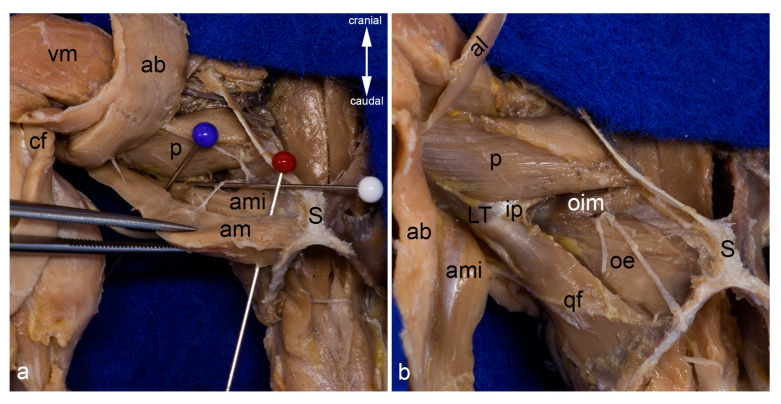
Deepest layers in the medial aspect of the thigh. The adductor magnus and adductor minimus muscles are shown from the medial aspect of the thigh. They were already separated by blunt dissection starting at the entrance of the twigs of the caudal branch of the obturator nerve (white pin). The cranial branch of this nerve was reflected cranially. The red pin was inserted between the origins of these two muscles, the blue pin near their insertions (**a**). The adductor magnus and adductor minimus muscles were detached from their origin and mobilized towards their insertion. Thus, the deep aspect of the pectineus, the quadratus femoris, part of the iliacus and psoas major muscles, as well as the obturator externus and obturator intermedius muscles can be observed from the ventral aspect (**b**). In both pictures, almost all superficial muscles were detached from their origin and reflected towards their insertion or totally removed. The thigh was abducted in the hip joint. ab = adductor brevis muscle, al = adductor longus muscle, am = adductor magnus muscle, ami = adductor minimus muscle, cf = caudofemoralis muscle, ip = iliacus and psoas major muscles, LT = lesser trochanter, oe = obturator externus muscle, oim = obturator intermedius muscle, p = pectineus muscle, S = pelvic symphysis, vm = vastus medialis muscle, qf = quadratus femoris muscle.

The adductor brevis muscle was detached from its origin. The caudal branch of the obturator nerve coursed on the deep aspect of this muscle. Thus, this structure was helpful to separate the adductor brevis muscle from the adductor magnus and minimus muscles (Figure 11a). Simultaneously, the semitendinosus, semimembranosus, and caudofemoralis muscles were carefully separated from the adductor muscles by blunt dissection. The muscular branches for the adductor brevis muscle from the cranial branch of the obturator nerve were cut to mobilize this muscle. Only then could both of its parts be seen in their entirety (Figure 5b and Figure 10b). Its genicular part was situated more superficially between the vastus medialis muscle proximal and the semimembranosus muscle distal. This part covered the distal portion of the caudofemoralis muscle, partly the attachment of the semimembranosus muscle, and the proximal portion of the tibial collateral ligament (Figure 10a,b). The femoral part of the adductor brevis muscle was situated more deeply with a wide field of insertion on the caudal aspect of the femur (see above). Its most distal fibers, which were attached to the medial and lateral supracondylar tuberosity, were in close contact to the fibrous capsule of the knee joint (Figure 5a). 

Deep to the adductor brevis muscle and to the caudal branch of the obturator nerve, the adductor magnus and minimus muscles were situated. In turn, these two muscles were in close contact with the obturator externus, quadratus femoris, pectineus, and iliopsoas muscles, but could be easily separated by blunt dissection (Figure 6c,d and Figure 11a). On first view, the adductor magnus muscle seemed to form one entity with the adductor minimus muscle. However, the two muscles could be separated constantly, starting at the entrance of their muscular branches from the caudal branch of the obturator nerve (Figure 11a). At their origin, the adductor magnus muscle was attached more ventrally and caudally, partly covering the adductor minimus muscle. Caudal to the adductor minimus muscle, the sciatic nerve and the caudofemoralis muscle descended (Figure 6b). 

The adductor magnus and minimus muscles were released from their origin. Their innervation by the caudal branch of the obturator nerve was transected to mobilize them towards their insertion. After this, almost the whole course of the obturator externus and intermedius muscles could be seen (Figure 11b). In addition, one can observe the pectineus muscle crossing the distal portions of the iliacus and psoas major muscles. The obturator intermedius muscle was situated deep to the pectineus muscle. The obturator externus muscle was partly covered by the quadratus femoris muscle, which originated from the outer aspect of the tabula of ischium, and coursed onto the distal portion of the intertrochanteric crest and the dorsal aspect of the lesser trochanter [27]. The common tendon of insertion of the obturator externus and intermedius muscles was attached in the trochanteric fossa. It was situated close to the common tendon of the gemellus and obturator internus muscles, which was attached on the medial aspect of the greater trochanter [27].

The obturator externus and intermedius muscles could be completely observed only by releasing the quadratus femoris and pectineus muscles (Figure 7). At first glance, the obturator externus and intermedius muscles seemed to be one individual. Nevertheless, they were separable along the branches of the obturator nerve exiting from the obturator canal. The obturator externus muscle was located caudally, and the obturator intermedius muscle cranially to the opening of the obturator canal. By careful separation of these two muscles, their innervation could be preserved. Each muscle received its own small twig directly from the stem of the obturator nerve (Figure 7d). Along the gap formed by the exit of the branches of the obturator nerve, the muscles could be completely separated to their proximal and distal ends. Harvesting them was carried out by first releasing their tendons of insertion from the trochanteric fossa. The belly of the obturator intermedius muscle could then be carefully peeled off from the bone. The release of the obturator externus muscle was more difficult due to the groove between the superficial edge of the bones surrounding the obturator foramen and the obturator membrane. The belly of the obturator externus muscle had to be carefully detached from the obturator membrane, and all parts of the bones surrounding the obturator foramen, including this groove (Figure 7b). During this procedure, the deep part of the muscle may be injured. 

## 4. Discussion

Due to its phylo- and ontogenesis, the adductor muscle group includes not only the adductor muscles proper, but also the gracilis muscle and the obturator externus muscle [1,2,8]. Although the pectineus muscle has a close topographical relationship due to its location and possible connections to other adductor muscles, its innervation indicates that it belongs to the iliopsoas group [1].

### 4.1. Gracilis Muscle

The gracilis muscle in the rats studied here always consisted of two completely separated muscles that were relatively broad. Partial or complete separation of the gracilis muscle seems to be a characteristic of rodents [1,6,8,12,17,19]. Therefore, in these species, the gracilis muscles in sum are considerably broader in comparison to the human gracilis muscle. Even in species in which it is not divided, such as ungulates, it is proportionally much broader [2]. In anthropoid apes, it is also much more powerful than in humans [10]. However, it is said to become longer with increasing opening angle of the hip and knee joint [2]. Therefore, in humans it is probably also extremely long due to the relatively long femoral shaft but has become slender and runs approximately parallel to the longitudinal axis of the femur [6].

In some species, the tendon of the gracilis muscle unites with the tendons of the sartorius and semitendinosus muscles near their insertion [2,9], as is also at least partially the case in humans through the formation of the so-called pes anserinus superficialis [32]. The rats studied here—like many other rodents [1,8,12,17]—lacked a sartorius muscle. It seems that this muscle disappears in these animals during embryogenesis, as observed during mouse embryogenesis [1]. The two gracilis muscles, therefore, only had a close relationship with the semitendinosus muscle, but were not connected to it. This could be because both the cranial gracilis and semitendinosus muscles were fleshy attached to the cranial border of the tibia. Only the caudal gracilis muscle had a tendon comparable to the human gracilis muscle. This tendon showed a tendency to unite with the cranial gracilis muscle. Due to the elongation for the upright gait, the very broad fleshy muscle attachments of the cranial gracilis and semitendinosus muscles in rats and other quadrupeds might then develop into the long tendons of insertion in humans. 

Although the rat has two gracilis muscles, they have distinct similarities with the single muscle in humans. The separation into two gracilis muscles seems to be a secondary process [1], since two-headed gracilis muscles can also occur in humans [33]. The origin of the rat’s cranial gracilis muscle with its broad aponeurotic tendon is like that of the human gracilis muscle [5], except that the origin of the rat’s cranial gracilis muscle is situated more cranially. The fleshy origin of the caudal gracilis muscle, on the other hand, is attached in a position analogous to that of the human muscle. Conversely, the caudal gracilis muscle in the rat has a similar tendon of insertion as in humans [5], whereas the cranial gracilis muscle has a broad fleshy insertion. Apparently, a broader muscle or a bipartition of the muscle is advantageous for the squat position, whereas due to the extension of the leg and the associated greater opening angle of the hip and knee joint [6], a long, narrow muscle achieves a better effect.

In summary, the two gracilis muscles in the albino rat were well distinguishable and constantly defined in all cases. They are topographically comparable to the human gracilis muscle, differing only in their division into two separate muscles. In addition, the length-to-width ratio is probably clearly altered due to the squatting position.

### 4.2. Adductor Muscles Proper

The adductor muscles proper are probably the most variable muscle group among all mammals and may sometimes cause difficulties in separating the individual parts. They can also partially fuse, or be split into even more parts [2]. Both features are also variably present in humans, where they may even fuse with the pectineus muscle [33]. This great variability can possibly be explained by phylogeny. In urodeles, turtles, and saurians, there is one adductor femoris muscle. In crocodilians and birds, this single muscle mass has largely split into two parts. In monotremes, there are usually three adductor muscles. Among mammals, the adductor muscles may be a single muscle mass or divided into two, three, or more individuals. The adductor femoris muscle or its parts are mostly innervated by the obturator nerve. In anurans, it is innervated completely, in saurians partially by the sciatic nerve [7]. According to Haines [24], only the adductor brevis muscle in mammals corresponds to the adductor femoris muscle of earlier forms. The adductor longus muscle is said to correspond to the flexor intercapitalis anterior muscle, and the adductor magnus muscle to the flexor intercapitalis posterior muscle of reptiles [24]. These two muscles insert between the two heads of the gastrocnemius muscle on the tibia. The flexor intercapitalis anterior muscle is innervated either by the obturator nerve or by the sciatic nerve. The flexor intercapitalis posterior muscle is innervated by the sciatic nerve. They were displaced onto the femur by the increase in size of the gastrocnemius muscle during phylogenesis and are thought to have changed their innervation in favor of the obturator nerve during this process. According to Haines, this is possible when the muscles adopt their “new” place early in embryogenesis [24,25]. In mouse embryogenesis, the adductor muscles begin to differentiate around the obturator nerve from a common ventral muscle mass from day 13 [1].

Also within domestic mammals, the adductor muscles proper are sometimes described as a uniform muscle mass [9], or two or three individuals are named but described in highly different ways [11,13,14,15,16]. According to Jauffrey [2], they form a powerful muscle complex that is supposed to be infinitely variably separable. Therefore, it should be difficult to identify, name, and homologize the individual muscles [19]. However, the naming of the adductor longus, brevis, and magnus muscles in comparative anatomy is always based on human anatomy [2]. This is despite the fact that the naming of the individual adductors is not conclusive either for human [3] or for comparative anatomy [6]. In this study, the naming of the individual adductors was consistently based on the topographical relationships of these muscles to the two branches of the obturator nerve and the innervation pattern of these branches, as in human anatomy [3,5]. The cranial and caudal branches in animals [13,28] correspond to the anterior and posterior branches in humans [29,31]. Following this rule, it was, therefore, possible to constantly separate and name the individual muscles. Thereby, it was shown that the individual muscles in the area of origin mostly behave similarly to humans, but there are sometimes clear differences in the area of the insertions. In the albino rats studied here, the adductor longus muscle was by far the smallest and shortest muscle. On the other hand, the adductor brevis muscle with its broad attachments reached furthest distally. The findings of this study were similar to the findings of some previous authors who used the same criteria for naming these muscles in other rodents [1,8,12,17]. On the other hand, several authors apparently used other criteria when naming the adductor muscles resulting in different descriptions, especially of the adductor brevis and magnus muscles [2,6,18,19,20]. In both human and comparative anatomy, only few authors described an adductor minimus muscle [3,17]. Sometimes it is only seen as part of the adductor magnus muscle [5]. In the rats examined in this study, an independent adductor minimus muscle could always be identified.

The adductor longus muscle was the smallest and most superficial of the adductor muscles in the rats studied here. Its narrow tendon of insertion was often fused with the much broader attachment of the pectineus muscle. The adductor longus muscle generally seems to be constant, as it is described similarly in other rodents and other species [2,6,8,12,17,18]. In other mammals including humans, it may be doubled or blended with other muscles [2,5]. In dogs, it is said to be completely fused with the pectineus muscle [11,34]. In the lesser bushbaby, it tends to fuse with the adductor magnus muscle [23]. In its location, it is similar to the human adductor longus muscle, but the proportions are exactly reversed: in humans, it originates with a rounded tendon and attaches broadly to the medial lip of the linea aspera [3,4]. These differences are probably due to the likewise reversed proportions of pelvic length and femoral length.

The adductor brevis muscle is partly difficult to compare with previous descriptions because they are very heterogeneous [2,6,8,12,17,18,19,20]. When starting dissecting the albino rats, the distal and bifurcated insertion of the adductor brevis muscle caused problems in homologizing this muscle. The main question was whether it was really a single muscle or consisted of two individuals. There is, in fact, a muscle in monotremes that splits off from the semimembranosus muscle, extending to the medial femoral condyle and even further proximally. This so-called praesemimembranosus muscle can be partially fused with an adjacent adductor muscle [7]. The praesemimembranosus muscle is thought to be innervated by either the sciatic nerve, the obturator nerve, or even both [24,25]. In this study, therefore, several attempts were made to separate the very strong adductor brevis muscle from its two insertion portions towards its origin. In fact, this would have led to an artificial splitting of the muscle. Furthermore, a praesemimembranosus muscle should arise from the ischium together with the semimembranosus muscle, which was not true for any part of the adductor brevis muscle observed here. Neither in domestic mammals [9,11] nor in other species [2,10] is the adductor brevis muscle described as it was observed in this study. Due to the lack of information on the position of the muscle in relation to the branches of the obturator nerve and the lack of proper illustrations, it was also not possible to verify whether the adductor brevis was mistaken for the adductor magnus muscle by some authors [2,6]. In some other rodents, the attachment behavior of the muscle was described very similarly to our findings [8,12]. Also, during mouse embryogenesis, the adductor brevis muscle migrates the furthest distally of all adductor muscles [1]. Ryan [17] even specifically named the two parts of the adductor brevis muscle (femoral and genicular parts), which were also adopted for this study. Also, in humans, the adductor brevis muscle tends to split partly, as it may consist of two bellies [3,4]. All these findings have finally confirmed the assignments of the muscle names chosen here. When comparing to the human condition, one just has to accept that the muscle named as the adductor brevis muscle of the rat—probably again due to the squatting position—extends much further distally than the human adductor brevis muscle. With this knowledge, the muscle and its two portions could be consistently dissected and identified. 

Since the adductor brevis muscle is much thicker in rodents, and extends far distally, it might be possible for parts of it to be incorporated into other muscles in humans. Thus, the portion of the femoral part that is attached to the lateral lip of the facies aspera and borders the adductor hiatus could possibly merge into the main part of the human adductor magnus muscle. It would be reasonable to assume that this part of the muscle is already innervated in the rat by the caudal branch of the obturator nerve. However, no double innervation of the adductor brevis muscle could be observed in the rats studied here. The genicular part of the adductor brevis muscle in rats is in immediate proximity to the vastus medialis muscle. Therefore, it would be predisposed to become the so-called pars obliqua of this muscle in humans. This distal portion of the vastus medialis muscle is even considered by some authors to be an independent muscle [35]. 

The adductor magnus muscle is also described very heterogeneously [2,9,11], or it is obviously confused with the adductor brevis muscle [2,6] as mentioned above. In the rats studied here, its distinction from the adductor brevis muscle was clear due to the position of the caudal branch of the obturator nerve, which coursed between the adductor brevis and adductor magnus muscles. The lateral portion of the femoral part of the adductor brevis muscle was attached to the lateral lip of the facies aspera in direct continuation to the adductor magnus muscle, but both muscles were always distinguishable. An independent adductor minimus muscle has neither been described in domestic mammals [9,11] nor in other species [1,2,8,12]. In primates, an adductor minimus may occasionally split off from the adductor magnus muscle [2,10], or it has not been described at all [23]. In the rats examined here, an adductor minimus muscle was always distinguishable, and could always be completely separated from the adductor magnus muscle by the entry of their nerve branches from the caudal branch of the obturator nerve. In rodents, only Ryan [17] described an adductor minimus muscle. However, his description differs from the findings in the rats studied here. Even in human anatomy, it is only rarely considered as an independent muscle [3,4,32]. Occasionally, it is described as part of the adductor magnus muscle [5]. The latter is perhaps due to the fact that sometimes the two muscles can be fused [33].

In humans, the adductor magnus muscle consists of two parts, which can occasionally be more or less separable [4,5]. Comparing this with the findings in the rat reported here, the major portion of the human adductor magnus muscle is likely to correspond not only to the adductor magnus muscle of the rat, but also to the lateral portion of the femoral part of the adductor brevis muscle (see above). In humans, the part known as the ischiocondylar portion is situated quite dorsally, courses almost vertically, and is innervated by the tibial nerve [3,5]. It is occasionally completely separable from the main part of the adductor magnus muscle [4,33]. It is apparently derived from a muscle of the ischiocrural (hamstring) group [2,8]. Whether this is the so-called praesemimembranosus muscle or the caudofemoralis muscle has been discussed for a long time [2,8,36]. In the rats studied here, a caudofemoralis muscle was always present (unpublished data). Further evidence for the rearrangement of the adductor magnus muscle in humans can be found in the comparison of the adductor hiatus. In the rats studied here, this opening was always bounded by the femoral part of the adductor brevis muscle and the distal part of the caudofemoralis muscle. Therefore, in humans, the former should correspond to that portion of the adductor magnus muscle proper, which borders the adductor hiatus medially. As discussed above, the caudofemoralis muscle of the rat is likely to correspond to the ischiocondylar portion of the adductor magnus muscle in humans, also because of its topographical relationship to the adductor hiatus. Strictly speaking, the ischiocondylar portion of humans is not an adductor muscle because of its location and innervation.

The adductor minimus muscle of the rats examined here is topographically comparable to that of humans. However, it is relatively thicker and longer, and its origin extended to the cranial pubic ramus in the examined rats, whereas in humans it only originates from the ischium and the inferior pubic ramus [3]. The site of insertion is also very similar. The position to other muscles and to the sciatic nerve also corresponds to that in humans. Probably because of the extended hip joint, it is relatively shorter in humans.

### 4.3. Obturator Externus and Intermedius Muscles

All the rats studied here not only had a very constantly developed obturator externus muscle, but also always a so-called obturator intermedius (or tertius) muscle. While the obturator externus muscle seems to be very consistently present throughout all species, the obturator intermedius muscle seems to be poorly known. In monotremes, turtles, and saurians, the obturator externus muscle always consists of two portions [7]. In some mammals (rodents, primates), it too tends to split [2,7]. The proper obturator externus muscle originates from the bony frame of the obturator foramen and inserts in the trochanteric fossa. In birds and some mammals, it enters the inside of the pelvis—pushing the obturator membrane in front of it—and is said to displace the origin of the obturator internus muscle [7]. This may also have been the case in the rats studied here, although the muscle did not actually enter the pelvis, but only occupied the complete depth of the frame of the obturator foramen. The obturator intermedius (tertius) muscle originates from the cranial pubic ramus and inserts on the lesser trochanter. Often the separation is incomplete and the two muscles are united at their insertion [2,7]. The latter was also the case in the rats studied here, as they were always attached together in the trochanteric fossa. The splitting into two muscles is always marked by the passage of the obturator nerve [2,7] which was confirmed by the present study. In addition, separate muscular branches for the two individuals could be consistently dissected.

In humans [5], anthropoid apes [10,23], domestic mammals [9,11], and rodents [1,6,8,12,17,18,19,20], this muscle complex is usually interpreted as a single muscle. Although the posterior branch of the obturator nerve is described as piercing the obturator externus muscle in humans [5], few authors see that the muscle actually consists of two portions in humans too [3,4]. The cranial bundle probably corresponds to the obturator intermedius muscle. Our own observations show that this muscle is—at least occasionally—also distinctive in humans. 

The obturator intermedius muscle is supposed to tend to unite with the pectineus muscle in primates and also in humans, which may explain the pectineus muscle’s double innervation [2]. In the rats studied here, these two muscles were situated completely parallel to each other, although they were clearly separated towards their insertion by the lesser trochanter. Should a fusion occur in other species, the obturator intermedius muscle would have to shift its insertion. Due to the changed angles in the upright position [6], this could well be possible in humans.

In sum, the muscle complex consisting of the obturator externus and intermedius muscles in the albino rat is topographically comparable to the human obturator externus muscle. In any case, further studies are needed to determine whether and to what extent the obturator intermedius muscle is present in humans and also in other species. Since it is rarely referred to as such, few anatomists are really familiar with it. 

## 5. Conclusions

As hypothesized, the muscles of the adductor muscle group in the albino rat (*Rattus norvegicus*) were constantly separable and could be clearly assigned to their names based on the selected topographical criteria. Therefore, our descriptions can be used to compare this muscle group with other species for future work. 

Despite the different resting posture and range of movements in the hip and knee joints between albino rats and humans, the topography of the adductor muscles is largely comparable between the two species. Differences are mainly found in the proportions and partly shifted areas of origin and insertion of some muscles. This is probably due to the longer but narrower pelvis and shorter femur of the rat compared to humans. The muscles of the albino rat adductor group may, therefore, be used as a model for further analyses (for example, muscle fiber typing or muscle spindle number) to provide initial evidence for these parameters in other species, including humans.

The research for this study has also shown that the muscles studied here are not completely understood and described in detail either in human anatomy or in comparative anatomy. Further research is needed, for example, on the occurrence and gross morphology of the obturator intermedius muscle in humans and other species.

## Figures and Tables

**Table 1:** Systematic anatomy of the adductor muscles in the albino rat.

Muscle	Origin	Insertion	Innervation
Cranial gracilis muscle	cranial and caudal pubic rami	cranial border of the tibia between tibial tuberosity and insertion of the semitendinosus muscle	cranial branch of the obturator nerve
Caudal gracilis muscle	ramus of ischium	medial surface of the tibia deep to the insertion of the cranial gracilis muscle	cranial branch of the obturator nerve
Adductor longus muscle	cranial and caudal pubic rami, ramus of ischium	medial lip of the facies aspera of the femur (fused with distal portion of insertion of pectineus muscle)	cranial branch of the obturator nerve
Adductor brevis muscle	cranial and caudal pubic rami	femoral part medial and lateral lip of facies aspera, medial and lateral supracondylar tuberosities	cranial branch of the obturator nerve
		genicular part medial femoral epicondyle, medial patellar retinaculum, medial tibial condyle	
Adductor magnus muscle	ramus of ischium	distal part of caudal aspect of the third trochanter, lateral lip of the facies aspera between adductor minimus (proximal) and adductor brevis (distal)	caudal branch of the obturator nerve
Adductor minimus muscle	cranial and caudal pubic rami (95% of cases), ramus of ischium (75% of cases)	caudal aspect of the third trochanter	caudal branch of the obturator nerve
Obturator externus muscle	cranial and caudal pubic rami, ramus of ischium, cranial margin of tabula of ischium, outer aspect of the obturator membrane	trochanteric fossa, fibrous capsule of the hip joint	obturator nerve (trunk)
Obturator intermedius muscle	cranial pubic ramus		obturator nerve (trunk)

For each muscle, the exact origin, insertion, and innervating nerves are summarized here. Within this muscle group, there were only rare remarkable variations, which are given in percentages.

## Data Availability

There are no data to share—all results are presented in this manuscript.

## References

[1] Jones C.L. (1979). The morphogenesis of the thigh of the mouse with special reference to tetrapod muscle homologies. J. Morphol..

[2] Jouffrey F.-K., Grassé P.-P. (1971). Musculature des membres. Traité de Zoologie. Anatomie, Systématique, Biologie. Tome XVI, Fascicule III.

[3] Frohse F., Fränkel M., Bardeleben K.V. (1913). Die Muskeln des menschlichen Beines. Handbuch der Anatomie des Menschen.

[4] Henle J. (1871). Handbuch der Systematischen Anatomie des Menschen. Erster Band. Dritte Abtheilung. Muskellehre.

[5] Williams P.L., Warwick R. (1980). Gray’s Anatomy.

[6] Howell A.B. (1926). Anatomy of the Wood Rat.

[7] Ribbing L., Bolk L., Göppert E., Kallius E., Lubosch W. (1938). Die Muskeln und Nerven der Extremitäten. Handbuch der Vergleichenden Anatomie der Wirbeltiere.

[8] Rinker G.C. (1954). The comparative myology of the mammalian genera *Sigmodon*, *Oryzomys*, *Neotoma*, and *Peromyscus* (Cricetinae), with remarks on their intergeneric relationships. Misc. Publ. Mus. Zool. Univ. Mich..

[9] Ellenberger W., Baum H. (1926). Handbuch der Vergleichenden Anatomie der Haustiere.

[10] Hepburn D. (1892). The comparative anatomy of the muscles and nerves of the superior and inferior extremities of the anthropoid apes: Part II. J. Anat. Physiol..

[11] König H.E., Liebich H.-G. (2020). Veterinary Anatomy of Domestic Animals.

[12] Stein B.R. (1986). Comparative limb myology of four arvicolid rodent genera (Mammalia, Rodentia). J. Morphol..

[13] Constantinescu G.M. (2018). Illustrated Veterinary Anatomical Nomenclature.

[14] Budras K.-D., Habel R.E. (2003). Bovine Anatomy.

[15] Budras K.-D., Sack W.O., Röck S., Horowith A., Berg R. (2011). Anatomy of the Horse.

[16] Evans H.E., de Lahunta A. (2013). Miller’s Anatomy of the Dog.

[17] Rayn J.M. (1989). Comparative myology and phylogenetic systematics of the Heteromyidae (Mammalia, Rodentia). Misc. Publ. Mus. Zool. Univ. Mich..

[18] Hebel R., Stromberg M.W. (1986). Anatomy and Embryology of the Laboratory Rat.

[19] Parsons F.G. (1894). On the myology of the sciuromorphine and hystricomorphine rodents. Proc. Zool. Soc. Lond..

[20] Parsons F.G. (1896). Myology of rodents. Part II. An account of the myology of the Myomorpha, together with a comparison of the muscles of the various suborders of rodents. Proc. Zool. Soc. Lond..

[21] Solomon L.B., Lee Y.C., Callary S.A., Beck M., Howie D.W. (2010). Anatomy of piriformis, obturator internus and obturator externus. Implications fot the posterior surgical approach to the hip. J. Bone Jt. Surg. Br..

[22] Yoo S., Dedova I., Pather N. (2015). An appraisal of the short lateral rotators of the hip joint. Clin. Anat..

[23] Stevens J.L., Mitton S., Edgerton V.R. (1972). Gross anatomy of hindlimb skeletal muscles of the *Galago senegalensis*. Primates.

[24] Haines R.W. (1934). The homologies of the flexor and adductor muscles of the thigh. J. Morphol..

[25] Haines R.W. (1935). A consideration of the constancy of muscular nerve supply. J. Anat..

[26] Bohensky F. (1986). Photo Manual and Dissection Guide of the Rat.

[27] Pretterklieber B., Pretterklieber M.L., Kerschan-Schindl K. (2022). Topographical anatomy of the albino rat’s ischiotrochanteric muscle group. Sci. Rep..

[28] International Committee on Veterinary Gross Anatomical Nomenclature (2017). Nomina Anatomica Veterinaria.

[29] Dauber W. (2007). Pocket Atlas of Human Anatomy Founded by Heinz Feneis.

[30] Dauber W. (2008). Feneis’ Bild Lexikon der Anatomie.

[31] Federative Committee on Anatomical Terminology (1998). Terminologia Anatomica—International Anatomical Terminology.

[32] Leonhardt L., Tillmann B., Töndury G., Zilles K. (1998). Rauber/Kopsch—Anatomie des Menschen: Lehrbuch und Atlas, Bd. 1 Bewegungsapparat.

[33] Bergmann R.A., Afifi A.K., Miyauchi R. (1996–2020). Illustrated Encyclopedia of Human Anatomic Variation. https://www.anatomyatlases.org/.

[34] Budras K.-D., McCarthy P.H., Fricke W., Richter R. (2007). Anatomy of the Dog.

[35] Smith T.O., Nichols R., Harle D., Donell S.T. (2009). Do the vastus medialis obliquus and vastus medialis longus really exist? A systematic review. Clin. Anat..

[36] Hill J.E. (1934). The homology of the presemimembranosus muscle in some rodents. Anat. Rec..

